# RAF dimer inhibition enhances the antitumor activity of MEK inhibitors in *K‐RAS* mutant tumors

**DOI:** 10.1002/1878-0261.12698

**Published:** 2020-05-18

**Authors:** Xi Yuan, Zhiyu Tang, Rong Du, Zhan Yao, Shing‐Hu Cheung, Xinwen Zhang, Jing Wei, Yuan Zhao, Yunguang Du, Ye Liu, Xiaoxia Hu, Wenfeng Gong, Yong Liu, Yajuan Gao, Zhiyue Huang, Zongfu Cao, Min Wei, Changyou Zhou, Lai Wang, Neal Rosen, Paul D. Smith, Lusong Luo

**Affiliations:** ^1^ Department of Discovery Biology BeiGene (Beijing) Co., Ltd. China; ^2^ Department of Pharmacology BeiGene (Beijing) Co., Ltd. China; ^3^ Program in Molecular Pharmacology Memorial Sloan Kettering Cancer Center New York NY USA; ^4^ Department of Biochemistry BeiGene (Beijing) Co., Ltd. China; ^5^ Global Statistics and Data Science BeiGene (Shanghai) Co., Ltd. China; ^6^ Department of Chemistry BeiGene (Beijing) Co., Ltd. China; ^7^ AstraZeneca CRUK Cambridge Institute Robinson Way UK; ^8^ External Innovation BeiGene, Ltd. San Mateo CA USA

**Keywords:** combination therapy, MEK inhibitor, RAF dimer inhibitor, RAS‐mutated cancer, synergy

## Abstract

The mutation of *K‐RAS* represents one of the most frequent genetic alterations in cancer. Targeting of downstream effectors of RAS, including of MEK and ERK, has limited clinical success in cancer patients with *K‐RAS* mutations. The reduced sensitivity of *K‐RAS*‐mutated cells to certain MEK inhibitors (MEKi) is associated with the feedback phosphorylation of MEK by C‐RAF and with the reactivation of mitogen‐activated protein kinase (MAPK) signaling. Here, we report that the RAF dimer inhibitors lifirafenib (BGB‐283) and compound C show a strong synergistic effect with MEKi, including mirdametinib (PD‐0325901) and selumetinib, in suppressing the proliferation of *K‐RAS‐*mutated non‐small‐cell lung cancer and colorectal cancer (CRC) cell lines. This synergistic effect was not observed with the B‐RAF^V600E^ selective inhibitor vemurafenib. Our mechanistic analysis revealed that RAF dimer inhibition suppresses RAF‐dependent MEK reactivation and leads to the sustained inhibition of MAPK signaling in *K‐RAS*‐mutated cells. This synergistic effect was also observed in several *K‐RAS* mutant mouse xenograft models. A pharmacodynamic analysis supported a role for the synergistic phospho‐ERK blockade in enhancing the antitumor activity observed in the *K‐RAS* mutant models. These findings support a vertical inhibition strategy in which RAF dimer and MEKi are combined to target *K‐RAS*‐mutated cancers, and have led to a Phase 1b/2 combination therapy study of lifirafenib and mirdametinib in solid tumor patients with *K‐RAS* mutations and other MAPK pathway aberrations.

## Graphical abstract

The reduced sensitivity of K‐RAS‐mutated cancer cells to MEK inhibitors (MEKi), such as selumetinib and mirdametinib, is associated with feedback phosphorylation of MEK by upstream RAF reactivation. RAF dimer inhibitors, such as lifirafenib and compound C, in combination with MEKi potently suppress RAF‐dependent MEK phosphorylation and lead to sustained inhibition of MAPK signaling and tumor growth.

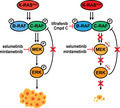

**Abbreviations**
CR: complete regression
CRC: colorectal cancer
EOHSA: excess over highest single agent
MAPK: mitogen‐activated protein kinase
MEKi: MEK inhibitors
NFA: negative feedback amplified
NSCLC: non‐small‐cell lung cancer
PR: partial regression
TGI: tumor growth inhibition
WT: wild‐type

## 1 Introduction

The RAS/RAF/MEK/ERK mitogen‐activated protein kinase (MAPK) cascade is one of the critical signaling pathways in regulating diverse cellular activities, including cell survival, growth, differentiation, and proliferation. In normal cells, stimulation of MAPK signaling occurs after binding of ligands to the membrane‐bound receptor tyrosine kinase. GTP‐bound RAS can then be activated, and subsequently promote the activation of RAF family proteins and transfer signals downstream through ERK phosphorylation (Dhillon *et al*., 2007). Gain‐of‐function mutations that lead to constitutive activation of the MAPK pathway are among the most common genetic alterations in human cancers. *RAS* and *RAF* are two of the most frequently mutated genes in promoting tumorigenesis (Holderfield *et al*., 2014; Schubbert *et al*., 2007). Mutated B‐RAF enzyme (e.g., B‐RAF^V600E^) phosphorylates and activates MEK1/2 in a RAS‐independent manner (Holderfield *et al*., 2014; Poulikakos *et al*., 2011). Such mutations have been detected in numerous human malignancies, including primary and metastatic melanomas, thyroid cancer, colorectal cancer (CRC), lung cancer, Barrett’s adenocarcinoma, breast cancer, cervical cancer, and hematologic cancer (Davies *et al*., 2002; Forbes *et al*., 2011). Oncogenic B‐RAF^V600E^ represents one of the druggable targets for cancer therapy based on preclinical target validation and epidemiology. First‐generation B‐RAF inhibitors including vemurafenib and dabrafenib have been approved for treating *B‐RAF^V600E^*‐mutated metastatic melanoma based on good clinical efficacy and acceptable safety (Ballantyne and Garnock‐Jones, 2013; Bollag *et al*., 2012).

In addition to *B‐RAF*, *RAS* mutations also lead to stimulus‐independent activation of the MAPK pathway and other downstream effectors in contributing to tumorigenesis. Oncogenic *RAS* mutations are commonly identified in human cancers. It is reported that *K‐RAS* or *N‐RAS* mutations account for 59% of pancreatic cancer, 39% of CRC, 30% of cancer of biliary tract, 17% of non‐small‐cell lung cancer (NSCLC), 15% of ovarian cancer, 15% of endometrium cancer, and 23% of blood cancer (Sanger Institute Cosmic Database) (Fernández‐Medarde and Santos, 2011; Forbes *et al*., 2011; Prior *et al*., 2012). Efforts in developing RAS‐directed molecular therapeutics have resulted in very few clinical candidates due to the challenges in selectively targeting the RAS GTPase with small molecule inhibitors (Gysin *et al*., 2011). Recently, two *K‐RAS^G12C^* mutant specific inhibitors, MRTX849 and AMG510, which could lock K‐RAS in an inactive GDP‐bound state, showed promising preclinical activity in inhibiting *K‐RAS^G12C^* mutant cell proliferation and xenograft tumor growth (Romero, 2020). The reported early clinical data showed promising efficacy signals and may represent a breakthrough in the treatment of *K‐RAS^G12C^* mutant tumors. Nevertheless, this approach has limitations in that its utility is restricted in *K‐RAS^G12C^* mutations. It is evident that there are still high unmet medical needs for oncogenic *K‐RAS* mutants beyond *K‐RAS^G12C^*. Attempts have been made in targeting MEK, which is situated downstream of RAS and RAF. However, inhibition of MEK alone so far has very limited success in patients with *K‐RAS*‐mutated cancers (Blumenschein *et al*., 2015; Caunt *et al*., 2015). Release of feedback inhibition in the RAF/MEK/ERK cascade is one of the causes of acute adaptive resistance to monotherapy of MEK inhibitors (MEKi) (Friday *et al*., 2008; Hatzivassiliou *et al*., 2013). It was reported that MEKi such as selumetinib and mirdametinib (PD‐0325901) can reactivate C‐RAF and increase the formation of RAF/MEK complexes, which in turn drives MAPK signaling and makes it less susceptible to MEK inhibition in *K‐RAS* mutant cells (Lito *et al*., 2014). It has been revealed both experimentally and mathematically that the three‐tiered kinase module of RAF/MEK/ERK integrated with negative feedback loops generates a biological circuit serving as a negative feedback amplified (NFA) (Fritsche‐Guenther *et al*., 2011; Sturm *et al*., 2010). Such NFA confers the robustness of the signaling pathway and renders it difficult to effectively inhibit aberrant cell signaling (Fritsche‐Guenther *et al*., 2011; Sturm *et al*., 2010). It is suggested that a combination of therapeutics that block multiple nodes within the feedback loops could weaken the NFA function (Sturm *et al*., 2010). Given that MEK itself is directly regulated and activated by RAF, which is subject to the negative feedback regulation of ERK, a vertical inhibition strategy based on the combination of RAF and MEKi should be a more effective approach for treating *K‐RAS* mutant tumors that are resistant to MEKi.

In wild‐type (WT) RAF‐expressing cells, RAF dimerization induced by upstream RAS is an important step and is required for MAPK pathway activation (Rajakulendran *et al*., 2009). The formation of a constitutive dimer of the B‐RAF mutant or transactivation of B‐RAF/C‐RAF heterodimer is the main mechanism that is involved in resistance to first‐generation B‐RAF inhibitors (Samatar and Poulikakos, 2014). The next‐generation RAF kinase inhibitors, represented by lifirafenib (BGB‐283), inhibits RAF family kinases including WT A‐RAF, B‐RAF, C‐RAF, and the B‐RAF^V600E^ mutant (Tang *et al*., 2015). BGB‐283 is currently in Phase I clinical trials in patients with *B‐RAF*‐ or *K‐RAS/N‐RAS*‐mutated solid tumors (Desai *et al*., 2016a). BGB‐283 single‐agent treatment was reported to lead to clinical benefit for patients with B‐RAF V600‐mutated melanoma, papillary thyroid cancer, and ovarian cancer. Antitumor activity also was observed in *K‐RAS*‐mutated NSCLC and endometrial cancer (Desai *et al*., 2017).

In this study, we tested a number of RAF and MEK inhibitor combinations. We showed that RAF dimer inhibitors including BGB‐283 and compound C when combined with MEKi yielded better antiproliferative activity in multiple *K‐RAS*‐mutated NSCLC and CRC cancer models. This combination synergy was not observed when vemurafenib, a first‐generation RAF inhibitor devoid of RAF dimer inhibition capability, was used for MEK combinations. We observed enhanced antitumor efficacy for RAF dimer and MEK inhibitor combination, represented by BGB‐283 and selumetinib, in NSCLC and CRC xenografts with *K‐RAS* mutations. The pharmacodynamic analysis further supported the role of synergistic phospho‐ERK blockade in enhancing the antitumor activity in the *K‐RAS* mutant models. There have been several reports suggesting RAF dimer or pan‐RAF inhibitor in combination with MEK inhibitor may be a strategy to target *K‐RAS*‐mutated cancers (Lamba *et al*., 2014; Whittaker *et al*., 2015; Yen *et al*., 2018). This report represents the first systematic comparison of different RAF and MEK combinations performed in a number of *in vitro* and *in vivo* K‐RAS models and sheds more light on the molecular mechanism underpinning this combination. These results support a new strategy to target *K‐RAS*‐mutated cancers in the clinic. This strategy is currently being tested in a Phase 1b study evaluating lifirafenib (BGB‐283) in combination with mirdametinib (PD‐0325901) in patients with advanced or refractory solid tumors harboring *RAS* mutations, *RAF* mutations, and other MAPK pathway aberrations.

## 2 Materials and methods

### 2.1 Reagents

BGB‐283, compound C, and pimasertib were synthesized in‐house and exceeded a purity of 99% as measured by proton nuclear magnetic resonance (HNMR), liquid chromatography–mass spectrometry (LC‐MS), and high‐performance liquid chromatography (HPLC). Compounds were purchased from following source: vemurafenib, WuXi AppTec (Shanghai, China); selumetinib, PD‐0325901, and trametinib, BioChemPartner (Shanghai, China); and RO5126766, Active Biochem (Kowloon, Hong Kong). Stock solutions of compounds were prepared in dimethyl sulfoxide. Antibodies used were obtained commercially from the following sources: anti‐B‐RAF (SC‐5248), Santa Cruz Biotechnology (Santa Cruz, CA, USA); anti‐C‐RAF (610152), BD Biosciences (San Jose, CA, USA); antibodies to MEK (9122), MEK1 (2352), phospho‐MEK1/2 (Ser217/221) (9154), ERK (4695), phospho‐ERK1/2 (Thr202/Tyr204) (4370), GAPDH (2118s), and anti‐rabbit IgG horseradish peroxidase (HRP)‐linked secondary antibody, Cell Signaling Technology (Danvers, MA, USA); and anti‐mouse IgG HRP‐linked secondary antibody (A0168), Sigma‐Aldrich (St. Louis, MO, USA). BALB/c nude mice (female) were purchased from Beijing HFK Bioscience Co., Ltd. (Beijing, China). All procedures involving animals were conducted in accordance with the Institutional Animal Care and Use Committee (IACUC) of BeiGene.

### 2.2 Cell culture

Calu‐6, SW1573, SW480, T84, SK‐LU‐1, DLD‐1, HCT8, NCI‐H2122, NCI‐H1299, and NCI‐H460 cells were purchased from American Type Culture Collection (ATCC, Manassas, VA, USA). LoVo, NCI‐H358, LS174T, and Calu‐1 cells were purchased from Cell Bank of Shanghai Institutes for Biological Sciences, Chinese Academy of Science (Shanghai, China). A549, HCC2998, and NCI‐H23 were kindly provided by National Institute of Biological Sciences (Beijing, China). Calu‐6, LS174T, LoVo, SW1573, T84, and SK‐LU‐1 cells were maintained in Dulbecco’s modified Eagle’s medium (DMEM; Gibco, Gaithersburg, MD, USA). A549, DLD‐1, HCT8, SW480, HCC2998, NCI‐H2122, NCI‐H23, NCI‐H1299, NCI‐H460, and NCI‐H358 cells were maintained in RPMI‐1640 (Gibco). Calu‐1 cells were maintained in McCoy’s 5A medium (Gibco). All growth media were supplemented with 10% FBS (Thermo Scientific, Waltham, MA, USA), 100 units·mL^−1^ penicillin (Gibco), and 0.1 mg·mL^−1^ streptomycin (Gibco). Cell lines were reinstated from frozen stocks and passaged no more than thirty times.

### 2.3 *In vitro* kinase assay

Compounds were tested for inhibition of RAF kinase activity in assays based on the time‐resolved fluorescence resonance energy transfer (TR‐FRET) methodology. RAF kinases were immunoprecipitated from HEK293 cells overexpressing WT B‐RAF, B‐RAF^V600E^, or C‐RAF. MEK1 (K97R) was used as a substrate for RAF kinases (Cisbio Bioassays, Codolet, France). The kinase was incubated with compounds for 1, 6, and 24 h at room temperature (RT), and ATP (final concentration at 1 mm) and kinase substrates were added to initiate the reaction. The reaction was stopped by an equal volume of stop/detection solution according to the manufacture's instruction (Cisbio Bioassays). Plates were sealed and incubated for 2 h, and the TR‐FRET signals (ratio of fluorescence emission at 665 nm over emission at 620 nm with excitation at 337 nm wavelength) were recorded on a PHERAstar FS plate reader (BMG Labtech, Ortenberg, Germany).

### 2.4 Cell‐based phospho‐ERK and phospho‐MEK detection assay

Cellular phospho‐ERK and phospho‐MEK were measured in assays based on the TR‐FRET methodology. Cells were seeded at 3 × 10^4^ per well of a 96‐well plate and were incubated at 37 °C for 16 h. Cells were then treated with a 10‐point titration of testing compounds. After compound treatment, culture medium was removed, 50 μL of lysis buffer (Cisbio) was added to each well, and plates were incubated at RT with 30 min of shaking. A total of 16 μL of cell lysate from each well was transferred to a 384‐well small volume white plate. Lysate from each well was incubated with 2 μL of Eu^3+^‐cryptate (donor)‐labeled anti‐MEK or anti‐ERK antibody (Cisbio) and 2 μL of D2 (acceptor)‐labeled anti‐phospho‐ERK or anti‐phospho‐MEK antibody (Cisbio) for 2 h at RT. FRET signals were measured using a PHERAstar FS reader (BMG Labtech).

### 2.5 siRNA transfection

siRNA against B‐RAF (25 nm) and C‐RAF (25 nm) was transfected into cells using Lipofectamine RNAiMAX Reagent (Invitrogen, Carlsbad, CA, USA). For each well of a six‐well plate, 5 µL Oligofectamine was diluted in 250 µL DMEM (Gibco) and incubated for 10 min at RT. While incubating, an appropriate amount of siRNA was diluted in 250 µL DMEM. The diluted Lipofectamine RNAiMAX solution was then added to the siRNA and gently mixed. After incubation for 20 min at RT, the siRNA–Lipofectamine RNAiMAX complexes were added dropwise to the cells. After incubation for 48 h, cells were then treated with 1 μm selumetinib for 1 h.

### 2.6 Proliferation assays

The antiproliferative activity of compounds in a panel of *RAS* mutant NSCLC and CRC cell line was determined using CellTiter‐Glo luminescent cell viability assay (Promega). The number of cells seeded per well of a 96‐well plate was optimized for each cell line to ensure logarithmic growth over the three‐day treatment period. Cells were incubated for 16 h and then treated with a 10‐point dilution series in duplicate. Following a 3‐day exposure to compounds, a volume of CellTiter‐Glo reagent equal to the volume of cell culture medium was added into each well. Mixture was mixed on an orbital shaker for 2 min to allow cell lysing, followed by 10‐min incubation at RT to allow development and stabilization of luminescent signal. Luminescent signal was measured using PHERAstar FS reader (BMG Labtech).

### 2.7 EOHSA analysis

Excess over Highest Single Agent (EOHSA) is a standard criterion for evaluating drug combinatorial effects on cell growth inhibition (Bachman *et al*., 2012; Borisy *et al*., 2003). EOHSA was used to analyze the excess inhibition effects produced by the drug combination over the larger effects produced by two single agents at corresponding concentrations. For analysis purpose, it is assumed that the log of the difference of each raw measurement/positive control and the negative control follows a normal distribution with different means but the same variance. The model is fitted by the maximum‐likelihood method, and the EOHSA for each dose combination is calculated by applying the fitted model to the EOHSA calculation formula. We want to identify the set of dose combinations with synergy effects, say *J*. For such purpose, we consider the hypothesis testing problem:

$$H_{0}:{\text{EOHSA}}_{\mathrm{ij}}=0\text{vs}.H_{a}:{\text{EOHSA}}_{\mathrm{ij}}>\;0,\;\text{for all dose combination}\left(i,j\right)\in J.$$

The hypothesis testing procedure described in Perone Pacifico *et al*. (2004) is applied to control the false discovery exceedance at 5%, where the false discovery exceedance is known as the probability that the percentage of false discovery in the dose combination with nonsynergy effects is over 5%. This procedure consists of two steps. First, we tested if considered dose combinations containing any dose combination with synergy effects at significance level 5%. Next, we selected a set of dose combinations with synergy effects as a subset of *J* such that the percentage of false discovery in the dose combination with nonsynergy effects is over 5%. SynergyBG, an in‐house data processing and analysis tool set, was developed to estimate the EC_50_s for a single compound and for combined treatment.

### 2.8 Immunoprecipitation and immunoblotting

After compound treatment, cells were harvested and lysed immediately in cell lysis buffer (Cell Signaling Technology) supplemented with protease inhibitor (Millipore, Hayward, CA, USA) and phosphatase inhibitor (Sigma). The protein concentration of lysates was determined using the Pierce BCA protein assay kit (Thermo Scientific). For immunoprecipitation, cell lysates were incubated with MEK1 antibody at 4 °C overnight following with incubation with protein G Sepharose beads at 4 °C for 2 h. Immune complex was washed extensively and eluted in SDS sample buffer. Proteins were separated by NuPAGE Novex 4–12% Bis‐Tris protein gels and transferred to nitrocellulose membranes using iBlot™ Dry Blotting System (Life Technologies, Carlsbad, CA, USA). Blots were blocked with TBSTM (50 mm Tris (pH 7.5), 150 mm NaCl, 0.1% Tween‐20, and 5% nonfat milk) at RT for 1 h and probed with indicated antibodies diluted in TBSTM. For reprobing, the membranes were incubated with stripping buffer (25 mm glycine, pH 2.0, 1% SDS) for 30–60 min at RT, rinsed twice with TBST for 10 min, and probed for other proteins. Antigen–antibody complexes were visualized using the chemiluminescent substrate (Millipore) and detected with ImageQuant LAS 4000 Mini digital imaging system (GE Healthcare, Milwaukee, WI, USA). The immunoblot were quantified using imagej software (NIH, Bethesda, MD, USA).

### 2.9 *In vivo* efficacy studies

All procedures involving animals were conducted in accordance with approved protocol from the IACUC of BeiGene. For Calu‐6 and HCT116 xenografts, each mouse was injected subcutaneously with 3 × 10^6^ cells in 200 μL PBS in the right front flank *via* a 26‐gauge needle. When the average tumor size reached ~ 140 mm^3^, animals were randomized to treatment groups and treated twice per day (BID) by oral gavage (p.o.) with vehicle [0.5% methylcellulose (MC) + 2% Tween‐80] alone, selumetinib (25 mg·kg^−1^), BGB‐283 (5–15 mg·kg^−1^), or combination of selumetinib (25 mg·kg^−1^) and BGB‐283 (2.5–5 mg·kg^−1^). BGB‐283 and selumetinib were formulated at the desired concentration as homogenous suspension in 0.5% (w/v) MC or 0.5% (w/v) MC + 2% Tween‐80 in purified water. Individual body weights and tumor volumes were determined twice weekly, and mice were monitored daily for clinical signs of toxicity for the duration of the study. Tumor volumes were calculated using the following formula: *V* = 0.5 × (*a* × *b* ^2^), where *a* and *b* are the long and short diameters of the tumor, respectively. Partial regression (PR) was defined as tumor volume smaller than 50% of the starting tumor volume on the first day of dosing for at least three consecutive measurements, and complete regression (CR) was defined as tumor volume < 14 mm^3^ for at least three consecutive measurements. Tumor growth inhibition (TGI) was calculated using the following formula: % growth inhibition = 100×[1 − (treated *t *− treated *t*_0_)/(placebo *t *− placebo *t*_0_)], where treated *t* represents tumor volume at day *t* in the treated group, treated *t*_0_ represents *d* tumor volume of the same treated group on the first day of treatment, placebo *t* represents placebo tumor volume day *t* in the control group, and placebo *t*_0_ represents *d* tumor volume of the same group on the first day of treatment. Statistical analysis was conducted using the Student *t*‐test. *P* < 0.05 was considered statistically significant.

### 2.10 *In vivo* pharmacodynamic assay

Frozen tumor tissues were homogenized in 500 µL lysis buffer in MP homogenization unit (FastPrep®‐24; MP Bio, Irvine, CA, USA). Lysates were then centrifuged at 18 929 ***g*** for 10 min at 4 °C to remove insoluble materials. The protein concentration was determined by BCA assay, and 2 µg protein lysates was used to measure phosphorylated ERK1/2 level by AlphaScreen® SureFire® p‐ERK1/2 assay (PerkinElmer, San Jose, CA, USA).

## 3 Results

### 3.1 BGB‐283 and compound C but not vemurafenib inhibit RAF dimers

**Fig. 1:**
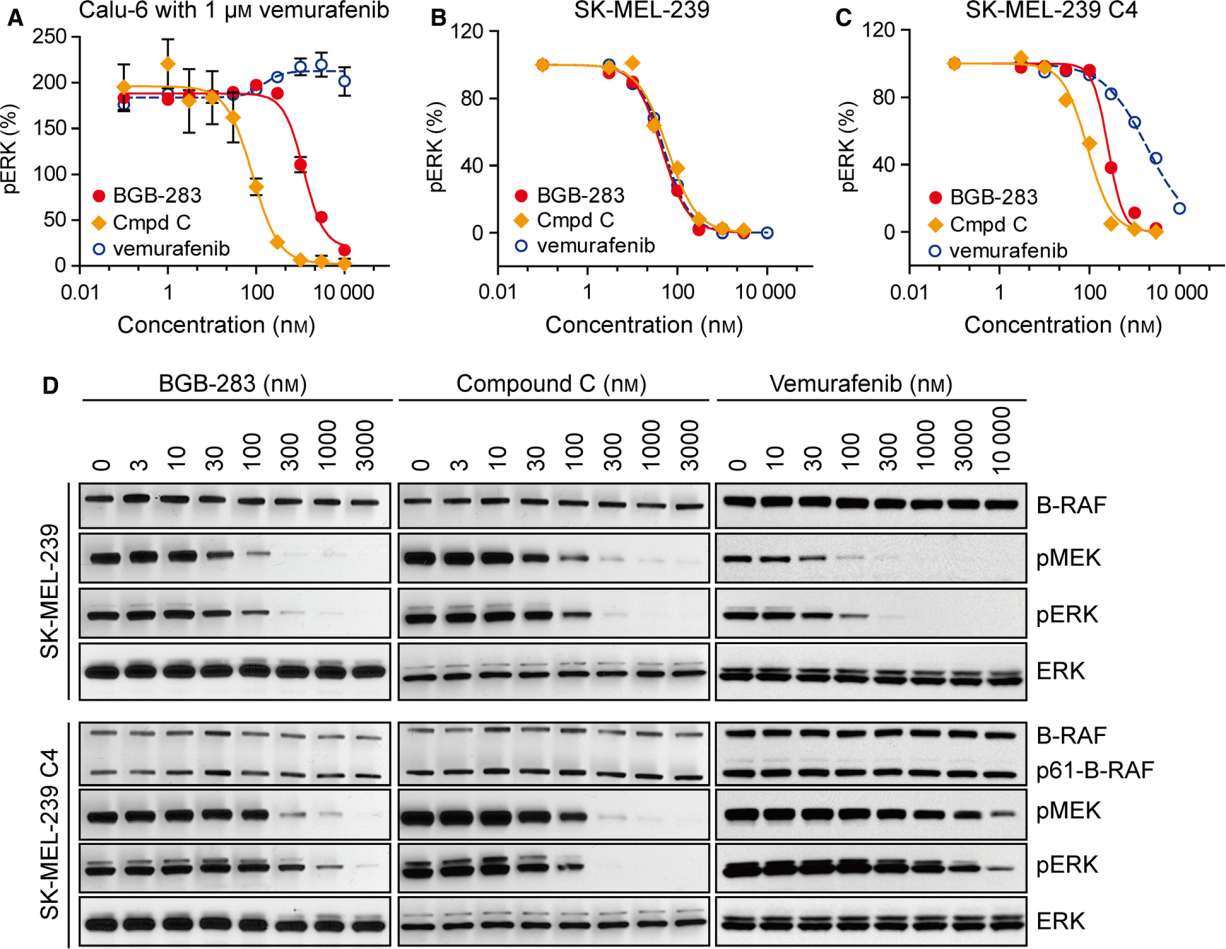
BGB‐283 inhibited RAF dimer activity. (A) Concentration–response curves of ERK phosphorylation were determined by HTRF assays in Calu‐6 cells treated with a combination of 1 µm vemurafenib and serial dilutions of BGB‐283, compound C, or vemurafenib for 1 h. Data are plotted as mean ± standard error of the mean (SEM) (*N* = 3). (B) Quantitative values of pERK levels normalized to ERK from (D) in SK‐MEL‐239 cells were plotted, and response curves were generated using GraphPad Prism (version 6.05). (C) Quantitative values of pERK levels normalized to ERK from (D) in SK‐MEL‐239 C4 cells were plotted, and response curves were generated. (D) Immunoblots probing for B‐RAF, pMEK, pERK, and ERK in parental SK‐MEL‐239 and subclone C4 cells treated with increasing concentration of BGB‐283, compound C, or vemurafenib for 1 h.

BGB‐283 and compound C are novel RAF kinase inhibitors with potent and reversible activities against WT A‐RAF, B‐RAF, C‐RAF, and B‐RAF^V600E^ (Tang *et al*., 2015). BGB‐283 and compound C showed potent and time‐dependent inhibition of WT B‐RAF, B‐RAF^V600E^, and WT C‐RAF at 1 mm ATP concentration, which is representative of intracellular ATP levels (Copeland *et al*., 2005) (Table 1). In comparison, vemurafenib only potently inhibited B‐RAF^V600E^ but not WT B‐RAF at physiologically relevant ATP concentration. This result is consistent with the previous reports demonstrating that first‐generation B‐RAF inhibitors including vemurafenib and dabrafenib are selective toward B‐RAF^V600E^ and inactive against WT B‐RAF (Karoulia *et al*., 2016; Poulikakos *et al*., 2010). To test the inhibitory activity of BGB‐283 on the RAF dimer, we used 1 µm vemurafenib to transactivate RAF dimer in Calu‐6 cells (Hatzivassiliou *et al*., 2010; Poulikakos *et al*., 2010). BGB‐283 and compound C inhibited vemurafenib‐induced ERK phosphorylation with an IC_50_ of 1.2 µm and 84.1 nm. No inhibitory effect was observed with vemurafenib (Fig. 1A). The RAF dimer activity was further evaluated in SK‐MEL‐239 cells stably expressing p61‐B‐RAF^V600E^, which forms RAS‐independent p61‐B‐RAF^V600E^ homodimer and leads to vemurafenib resistance (Poulikakos *et al*., 2011). In SK‐MEL‐239 cells harboring *B‐RAF^V600E^* mutation, BGB‐283, compound C, and vemurafenib all potently inhibited ERK phosphorylation (Fig. 1B,D). While p61‐B‐RAF^V600E^‐expressing SK‐MEL‐293 C4 cells were resistant to vemurafenib, BGB‐283 and compound C demonstrated strong concentration‐dependent inhibitory effect on phospho‐ERK in p61‐B‐RAF^V600E^‐expressing SK‐MEL‐293 C4 cells with an IC_50_ of 258 nm and 89.7 nm, respectively (Fig. 1C,D). In a recent publication, Yao et al. showed that BGB‐283 inhibited the second unoccupied site of LGX818‐half‐bound RAF dimers with good potency compared to other RAF inhibitors (Yao *et al*., 2015). In addition, BGB‐283 and compound C induced much less activation of ERK signaling and dose‐dependently inhibited ERK phosphorylation (Tang *et al*., 2015). All these findings demonstrate that BGB‐283 and compound C, unlike vemurafenib, inhibit RAF dimer in *K‐RAS*‐mutated cancer cells and in melanoma cells expressing the p61‐B‐RAF^V600E^ dimer.

**Table 1:** Time‐dependent inhibition of BGB‐283 and compound C against WT B‐RAF, V600E B‐RAF, and WT C‐RAF at 1 mm ATP. Compounds were pre‐incubated with immunoprecipitated enzyme and 1 mm ATP for 1, 6, or 24 h before 1 mm ATP and 2 × MEK were added to initiate the reaction.

	BGB‐283 IC_50_ (nm)			Compound C IC_50_ (nm)			Vemurafenib IC_50_ (nm)		
Pre‐incubation time	1 h	6 h	24 h	1 h	6 h	24 h	1 h	6 h	24 h
WT B‐RAF	2312	307	38	24	3.5	1.2	> 5000	> 5000	3893
V600E B‐RAF	49	22	12	1.6	0.44	0.31	40	62	165
WT C‐RAF	122	24	6.7	2.4	0.81	0.93	15	8.6	23

### 3.2 BGB‐283 but not vemurafenib enhances the inhibitory effect of MEKi selumetinib in *K‐RAS*‐mutated cancer cells

**Fig. 2:**
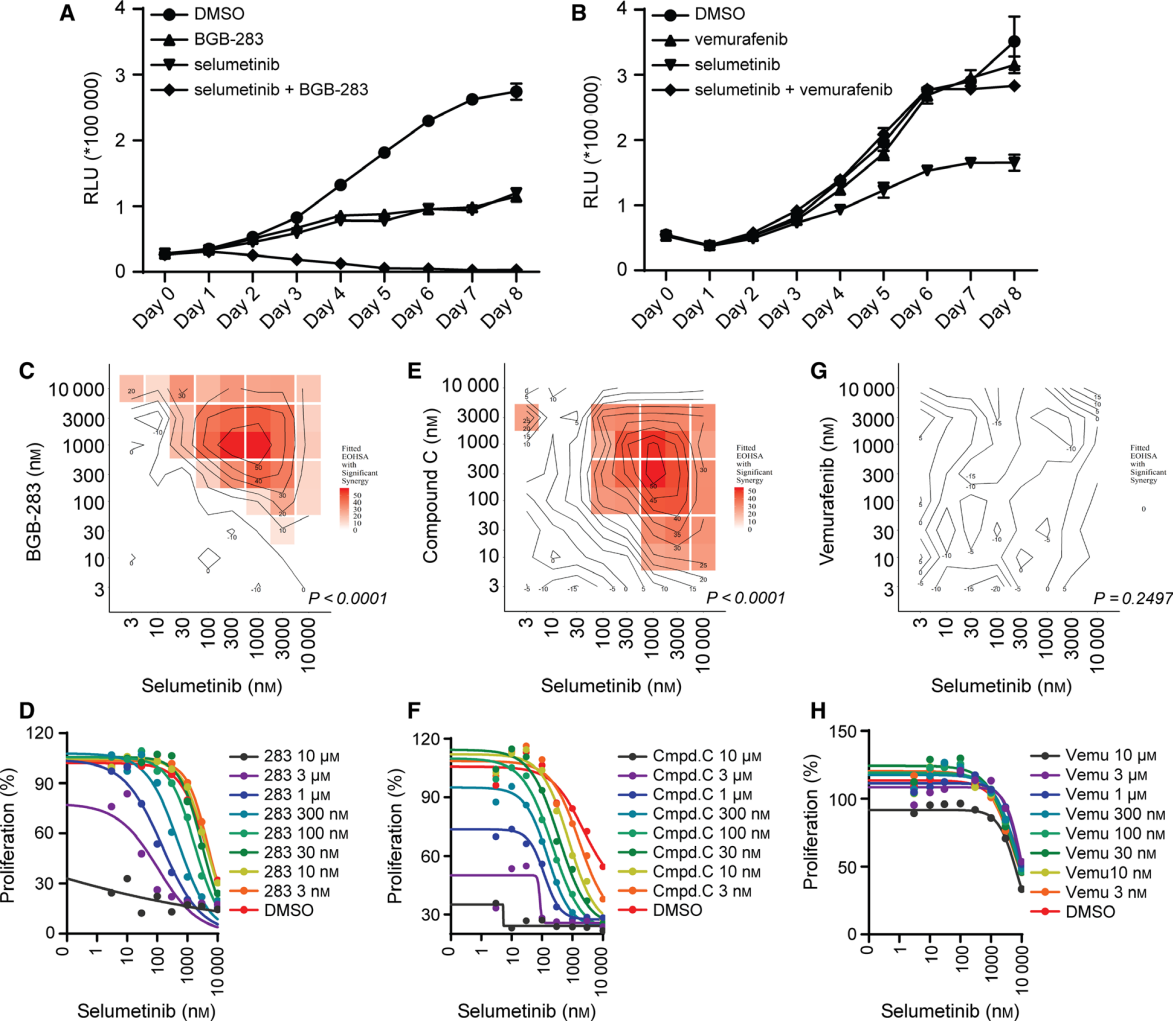
Combinatorial effect of RAF dimer inhibitors and selumetinib on the proliferation of Calu‐6 cells. Cell counts of Calu‐6 cells after the indicated days of treatment with (A) 1 µm selumetinib and 1 µm BGB‐283, or (B) 1 µm selumetinib and 1 µm vemurafenib as single agent or in combination. Data are plotted as mean ± standard deviation (SD) (*n* = 3). The effect of combining selumetinib and (C & D) BGB‐283, (E & F) compound C, or (G & H) vemurafenib was evaluated by EOHSA analysis. The considered dose combinations with synergy found by Pacifico’s approach at significance level 0.05 are highlighted.

We evaluated the inhibitory effect of combining BGB‐283 and MEKi selumetinib on the growth of *K‐RAS*‐mutated cancer cells. We observed that BGB‐283, but not vemurafenib, can potentiate the antiproliferative activity of selumetinib in Calu‐6 cells, which harbors *K‐RAS^Q61K^* mutation (Fig. 2A,B). The synergistic effect of the BGB‐283 and selumetinib combination was assessed in Calu‐6 cells using EOHSA analysis (Borisy *et al*., 2003). Various concentrations of BGB‐283 were combined across a range of concentrations of selumetinib to generate a 9 × 9 matrix. Each combination was then scored to identify antiproliferative effects that were greater than the effect of each individual component. For each dose combination, a model‐based EOHSA was calculated and hypothesis for synergy was tested under controlled false discovery exceedance (Perone Pacifico *et al*., 2004). In general, *P*‐values of Pacifico’s approach < 0.05 and maximal reduction of EC_50_ greater than fivefold are considered to have synergistic effect. In this study, selumetinib single agent showed marginal inhibitory effect on Calu‐6 cell growth with an EC_50_ of 3.4 μm. The addition of BGB‐283 decreased the EC_50_ value with a maximal > 100‐fold decrease to 30 nm for selumetinib in the presence of 3 µm BGB‐283 (Fig. 2D, Table 2). This combination also yielded *P*‐value < 0.0001 in the statistical model of EOHSA analysis, supporting the notion that BGB‐283 synergizes with selumetinib in suppressing Calu‐6 proliferation (Fig. 2C, Table 2). Similarly, compound C and selumetinib also show synergistic effect in inhibiting Calu‐6 cell proliferation, with a *P*‐value < 0.0001 and maximum IC_50_ shift for 28‐fold (Fig. 2E,F). In contrast, the vemurafenib and selumetinib combination did not show synergistic effect. The *P*‐value is 0.2497, and EC_50_ shift is within onefold (Fig. 2G,H). We expanded this analysis to a large panel of NSCLC and CRC cell lines harboring *K‐RAS* or *N‐RAS* mutations. Of interest, BGB‐283 significantly lowered the EC_50_ of selumetinib in twelve out of sixteen *K/N‐RAS* mutant NSCLC and CRC cell lines, with maximum EC_50_ difference ranging from 9‐ to over 100‐fold (Table 2). Additionally, we assessed combination effect of BGB‐283 and selumetinib in cell lines with WT RAF/RAS and without any known MAPK pathway abnormalities. In all three cell lines tested including HEK293, OUMS‐23, and NCI‐H209, BGB‐283/selumetinib combination did not show increased cytotoxic effect (Fig. S1). These data suggested that BGB‐283 exhibited a synergistic antiproliferative effect in combination with selumetinib selectively in *RAS* mutant cancer cells.

**Table 2:** BGB‐283 and selumetinib synergistically inhibited the proliferation of multiple NSCLC and CRC cell lines harboring *K/N‐RAS* mutations. No shift: Less than twofold EC_50_ shift was detected by combining BGB‐283 and various MEKi in the indicated cell lines.

Cell line	RAS mutation	EOHSA		Maximum EC_50_ shift for selumetinib
		*P*‐value*	Percentage^a^	
NSCLC:				
Calu‐6	K‐RAS^Q61K^	< 0.0001	0.45	**> 100‐fold ↓**
A549	K‐RAS^G12S^	< 0.0001	0.16	**77‐fold ↓**
NCI‐H2122	K‐RAS^G12C^	< 0.0001	0.30	**17‐fold ↓**
NCI‐H23	K‐RAS^G12C^	< 0.0001	0.25	**19‐fold ↓**
SW1573	K‐RAS^G12C^	< 0.0001	0.20	**> 100‐fold ↓**
NCI‐H358	K‐RAS^G12C^	< 0.0001	0.33	**9‐fold ↓**
NCI‐H1299	N‐RAS^Q61K^	< 0.0001	0.41	4‐fold **↓**
Calu‐1	K‐RAS^G12C^	0.0669	0.00	9‐fold ↓
SK‐LU‐1	K‐RAS^G12D^	< 0.0001	0.14	No shift
CRC:				
LS174T	K‐RAS^G12D^	0.0048	0.11	**18‐fold ↓**
LoVo	K‐RAS^G13D^	< 0.0001	0.22	**30‐fold ↓**
T84	K‐RAS^G13D^	0.0001	0.06	**19‐fold ↓**
DLD‐1	K‐RAS^G13D^	< 0.0001	0.20	**> 100‐fold ↓**
HCC2998	K‐RAS^A146T^	< 0.0001	0.23	**34‐fold ↓**
HCT8	K‐RAS^G13D^	0.0082	0.05	**9‐fold ↓**
SW480	K‐RAS^G12V^	0.9983	0.00	No shift

EC50 shift > fivefold is considered as significant in terms of combination synergy. They are highlighted in bold.

^a^ The percentage of the considered dose combinations with synergy found by Pacifico’s approach at significance level 0.05.

* *P*‐values of Pacifico’s approach for the hypothesis that at least one of the considered dose combinations has synergy per EOHSA.

### 3.3 RAF dimer inhibition enhances the inhibitory effect of MEKi in *K‐RAS*‐mutated cancer cells

**Fig. 3:**
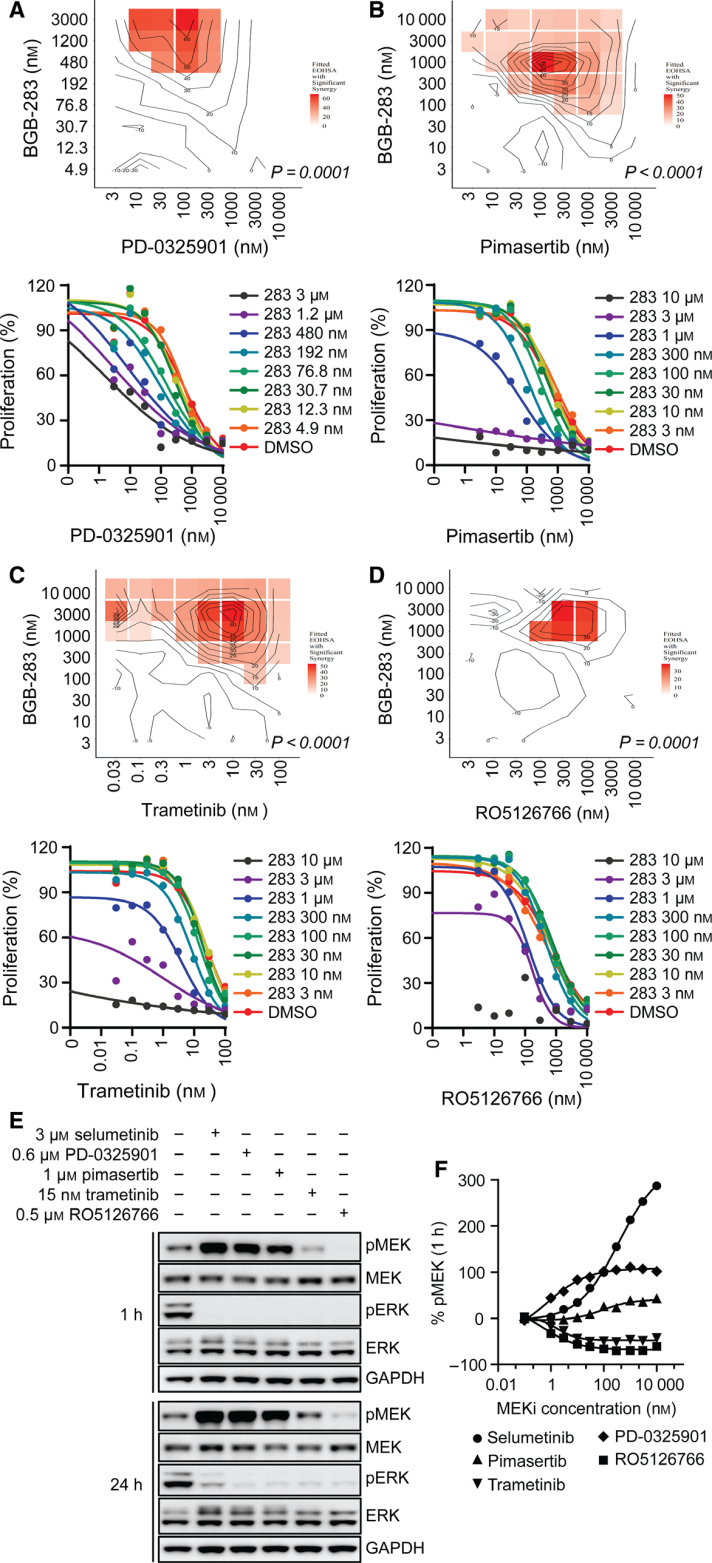
Effect of different allosteric MEKi combined with BGB‐283 and their impact on the MEK/ERK pathway in Calu‐6 cells. The antiproliferative effect of combining BGB‐283 and (A) PD‐0325901, (B) pimasertib, (C) trametinib, or (D) RO5126766 was evaluated by EOHSA analysis. The considered dose combinations with synergy found by Pacifico’s approach at significance level 0.05 are highlighted. (E) Immunoblot for pMEK, MEK, pERK, ERK, and GAPDH in Calu‐6 cells after 1‐ and 24‐h treatment with DMSO (‐), selumetinib, PD‐0325901, pimasertib, trametinib, and RO5126766 at the indicated concentrations. (F) Dose–response curve of MEK phosphorylation was determined by HTRF assay after 1‐h treatment of serial dilutions of different MEKi in Calu‐6 cells.

Previous studies revealed that MEKi such as selumetinib and mirdametinib (PD‐0325901) have limited activity in *K‐RAS* mutant tumors due the reactivation of C‐RAF by releasing the feedback inhibition from ERK (Lamba *et al*., 2014; Lito *et al*., 2014). Instead, trametinib and RO5126766 could reduce C‐RAF‐mediated MEK activation either by promoting the dissociation of RAF/MEK complexes or by preventing MEK phosphorylation by C‐RAF due to specific interaction within the activation segment (Lito *et al*., 2014). Given the strong synergistic effect observed for the BGB‐283 and selumetinib combination, we further investigated the ability of BGB‐283 to enhance the antiproliferative activities of other MEKi, including PD‐0325901, pimasertib, trametinib, and RO5126766, in *K/N‐RAS* mutant cell lines. The same method described above was used to generate 9 × 9 dose matrices and determine the growth inhibition. Each combination was then calculated for synergistic effect using EOHSA analysis. In Calu‐6, BGB‐283 was found to effectively enhance the antiproliferative activity of PD‐0325901 and pimasertib, with maximum EC_50_ shift for 59‐fold and 12‐fold in the presence of 3 and 1 µm BGB‐283, respectively (Fig. 3A,B, Table S1B,C). Similar synergistic effects were also observed for these two MEKi in combination with BGB‐283 in a number of other *K/N‐RAS* mutant NSCLC and CRC cell lines (Table S1A,B). Interestingly, less IC_50_ shift was observed for BGB‐283 in combination with trametinib and RO5126766 in Calu‐6 cells (Fig. 3C,D). We further expanded BGB‐283 and RO5126766 combination into eight NSCLC cell lines harboring *K/N‐RAS* mutations. BGB‐283 only showed limited effect in enhancing RO5126766 activity in these cells (Table S1C).

The effect of different MEKi on the MAPK signaling was further investigated in Calu‐6 cells by monitoring MEK and ERK phosphorylation. As shown in Fig. 3E,F, selumetinib, PD‐0325901, and pimasertib treatment at their respective EC_50_s effectively reduced pERK but stimulated a robust MEK phosphorylation through feedback activation within 1 h. The induction of MEK phosphorylation was more noticeable after 24 h (Fig. 3E). Knocking down endogenous B‐RAF or C‐RAF was found to abrogate selumetinib‐induced MEK phosphorylation in Calu‐6 cells (Fig. S2A). C‐RAF knockdown showed more prominent effect, suggesting that C‐RAF plays the major role in driving the MAPK signaling in *K‐RAS* mutant cells (Fig. S2A), which agreed with earlier findings (Blasco *et al*., 2011; Lito *et al*., 2014). In comparison, trametinib and RO5126766 potently inhibited MEK and ERK phosphorylation (Fig. 3E,F) and showed minimal pMEK upregulation. Trametinib and RO5126766 are weaker inducers of MEK feedback activation by either promoting the dissociation of RAF/MEK complexes or stabilizing the inactive interaction between MEK and RAF (Fig. S2B) (Lito *et al*., 2014). Herein, we showed that the combination effects of BGB‐283 and MEKi differed and were positively correlated with pMEK levels induced by MEK inhibitor treatment as single agent. Selumetinib, PD‐0325901, and pimasertib, which induced strong upregulation of pMEK through RAF activation, exhibited high synergistic effect when combined with BGB‐283. In contrast, BGB‐283 had diminished effect when combined with trametinib and RO5126766, which had weaker feedback activation of pMEK.

### 3.4 RAF dimer inhibition sequesters MEKi‐induced pMEK upregulation

**Fig. 4:**
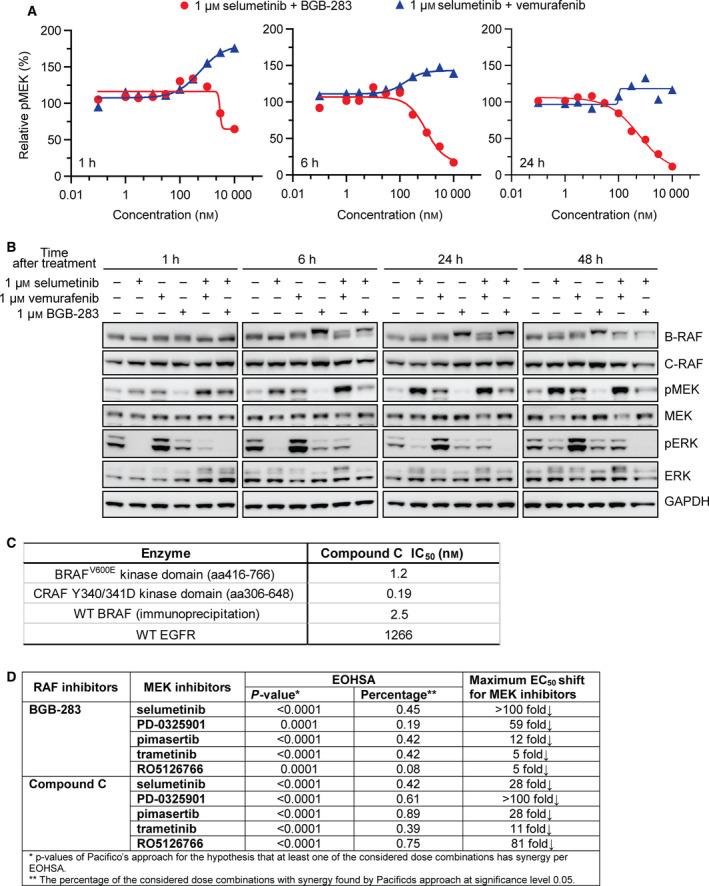
Combination of BGB‐283 and selumetinib effectively inhibited selumetinib‐induced pMEK accumulation and led to sustained pERK reduction. (A) Dose–response curve of MEK phosphorylation in Calu‐6 cells treated with 1 µm selumetinib combined with increasing concentrations of BGB‐283 or vemurafenib for 1, 6, and 24 h. (B) Immunoblotting of B‐RAF, C‐RAF, pMEK, MEK, pERK, and ERK in Calu‐6 cells incubated with 1 µm selumetinib alone or combined with 1 µm BGB‐283 or 1 µm vemurafenib after 1‐ to 48‐h treatment. (C) Biochemical activity of compound C against WT B‐RAF, B‐RAF^V600E^, C‐RAF, and EGFR. (D) Comparison of synergistic effect of MEKi with BGB‐283 or compound C from *P*‐value, the percentage of dose combination with synergy, and maximum EC_50_ shift in Calu‐6 cells.

It was previously reported that blocking MEKi‐induced pMEK upregulation could allow more effective inhibition of ERK phosphorylation in *K‐RAS*‐mutated cells (Hatzivassiliou *et al*., 2013; Lito *et al*., 2014). Selumetinib was found to trigger the time‐dependent increase in B‐RAF/C‐RAF complexes, suggesting the feedback activation of the pathway required RAF dimers (Lamba *et al*., 2014). Since BGB‐283 was shown to inhibit RAF dimer activity, we hypothesized that BGB‐283 could inhibit MEKi‐induced MEK phosphorylation and therefore improve the potency of the latter in inhibiting *K‐RAS*‐mutated cancer cells. The effect of BGB‐283 on a selumetinib‐mediated MEK phosphorylation was investigated in Calu‐6 cells. BGB‐283 was found to inhibit 1 μm selumetinib‐induced MEK phosphorylation in a time‐dependent manner, with IC_50_ of 2.9, 0.9, and 0.6 μm at 1‐, 6‐, and 24‐h treatment, respectively (Fig. 4A). In contrast, vemurafenib did not inhibit but rather induced MEK phosphorylation under the same conditions (Fig. 4A). In addition, combination treatment of BGB‐283 and selumetinib led to sustained inhibition of both MEK and ERK phosphorylation for up to 48 h (Fig. 4B). The synergistic effect between BGB‐283 and MEKi should be dependent on the RAF dimer activity of BGB‐283, rather than its anti‐EGFR activity (Tang *et al*., 2015), because compound C, a stronger RAF dimer inhibitor with minimal anti‐EGFR activity, showed better synergistic effect and further reduced antiproliferation EC_50_s of MEK single agent in Calu‐6 cells (Fig. 4C,D). These findings supported the notion that BGB‐283 prevented MEKi‐induced MAPK feedback signaling through RAF dimer inhibition and improved the antitumor activity of MEKi in *K‐RAS*‐mutated cancers.

### 3.5 Combination of BGB‐283 and selumetinib shows improved efficacy in xenograft models harboring *K‐RAS* mutations

**Fig. 5:**
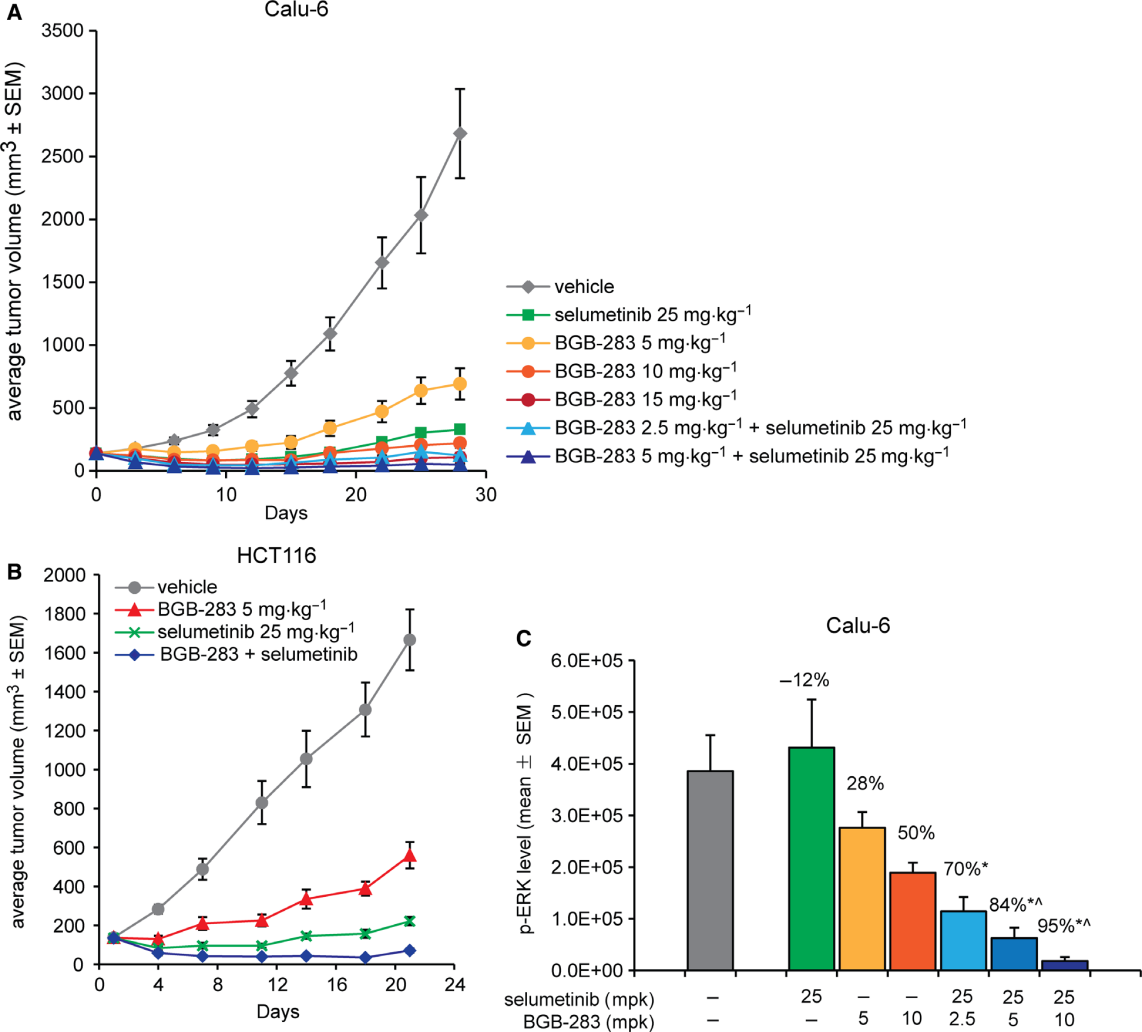
Combination of BGB‐283 and selumetinib exhibited enhanced antitumor activity in human CRC and NSCLC xenograft models bearing *K‐RAS* mutations. (A) Calu‐6 tumor cells (3 × 10^6^) were implanted subcutaneously in female BALB/c nude mice. When the tumors reached a mean volume of ~ 140 mm^3^ in size, mice were randomly allocated and treated as indicated. Data are presented as mean tumor volume ± SEM in each group (*N* = 9). (B) HCT116 tumor cells (3 × 10^6^) were implanted subcutaneously in female BALB/c nude mice. When the tumors reached a mean volume of ~ 140 mm^3^ in size, mice were randomly allocated and treated as indicated by oral gavage. Data are presented as mean tumor volume ± SEM in each group (*N* = 8). (C) PD analysis of pERK levels in tumor tissues at 12 h after the fifth dosing. Data are presented as mean ± SD of four animals in each group. The percentage of p‐ERK inhibition compared to the control is noted on the top of each group. Student’s *t*‐test was used to determine the statistical difference. *P < 0.05 vs. vehicle control; ^^^ *P* < 0.05 vs. corresponded selumetinib single treatment.

The combination effect of BGB‐283 and selumetinib was examined in a subcutaneous Calu‐6 NSCLC xenograft model. Both selumetinib and BGB‐283 as single agent resulted in detectable TGI (Fig. 5A). BGB‐283 alone at dosage ranging from 5 to 15 mg·kg^−1^ BID led to a dose‐dependent tumor regression. BGB‐283 treatment at 10 and 15 mg·kg^−1^ BID achieved 22% and 78% of PR, respectively (Table S2). However, a severe effect on body weight loss was detected along with better objective responses at higher dosages (Fig. S3). On the other hand, single treatment of selumetinib at 25 mg·kg^−1^ BID resulted in strong TGI (93%) on day 28, but with no objective tumor response (PR or CR) observed (Fig. 5A and Table S2). Combination treatments of selumetinib and BGB‐283 demonstrated enhanced antitumor efficacy over each single agent alone (Fig. 5A). Combination of selumetinib (25 mg·kg^−1^ BID) and all tested doses of BGB‐283 (2.5, 5 mg·kg^−1^ BID) induced tumor regression and resulted in 88% and 100% overall response rate (ORR, PR + CR) (Table S2). There was no significant effect on body weight in both combination treatment groups (Fig. S3). Drug exposure of both BGB‐283 and selumetinib was not significantly altered when used in combination, suggesting there was no drug–drug interaction between the two compounds (data not shown).

The synergistic antitumor activity of BGB‐283 and selumetinib could also be detected in a HCT116 CRC xenograft model that harbors a *K‐RAS* mutation (Fig. 5B). Selumetinib and BGB‐283 only showed limited efficacy in suppressing tumor growth when used as single agents (Fig. 5B). Following daily oral administration at well‐tolerated doses, BGB‐283 alone yielded 72% of TGI and selumetinib resulted in 94% of TGI with 13% of PR on day 21 (Table S2). Combination of BGB‐283 (5 mg·kg^−1^) and selumetinib (25 mg·kg^−1^) synergistically enhanced the antitumor activities of both compounds and achieved > 100% TGI and 88% PR on day 21 (Fig. 5B, Table S2). In all treatment groups, no significant effect on body weight was observed throughout the study (Fig. S3).

Pharmacodynamic studies were further conducted in Calu‐6 xenograft model to determine whether the improved tumor suppression of combining selumetinib and BGB‐283 corresponded with an effective inhibition of MAPK signaling. Tumors harvested at 12 h after the fifth dosing were lysed and subjected to measure ERK phosphorylation using the AlphaScreen assay. Selumetinib alone resulted in a 12% increase in ERK1/2 phosphorylation relative to the vehicle control (Fig. 5C). Single‐agent BGB‐283 treatment at 5 and 10 mg·kg^−1^ resulted in moderate reduction of pERK levels for 28% and 51%, respectively. In comparison, combination of selumetinib (25 mg·kg^−1^) with increasing doses of BGB‐283 at 2.5, 5, and 10 mg·kg^−1^ resulted in significant inhibition of ERK phosphorylation at 70%, 84%, and 95% (*P* < 0.05 vs. vehicle control), respectively (Fig. 5C). In summary, these data demonstrated the synergistic effect of BGB‐283 and selumetinib combination not only *in vitro* but also in *in vivo* models harboring *K‐RAS* mutations.

## 4 Discussion

The RAS/RAF/MEK/ERK signaling pathway is frequently dysregulated in multiple cancer types. Genetic mutations in *K‐RAS* and *B‐RAF* are two major causes of the pathway hyperactivation that leads to tumorigenesis. Inhibitors that selectively target mutated B‐RAF such as vemurafenib and dabrafenib have achieved high response rates and been approved for the treatment of melanoma patients with B‐RAF^V600E^ mutation (Ballantyne and Garnock‐Jones, 2013; Bollag *et al*., 2012). Although *K‐RAS* mutations are frequently observed in many cancers, effective targeted therapies are still lacking in patients with *K‐RAS* mutations. The recent development of K‐RAS^G12C^ covalent inhibitors showed promising early clinical activities and will inspire other approaches for targeting *K‐RAS* mutations beyond G12C. Unlike *B‐RAF^V600E^* mutant tumors in which mutated B‐RAF signals as a RAS‐independent monomer, *K‐RAS* mutant tumors cells with WT RAF activate the MAPK pathway through RAS‐dependent RAF dimerization (Freeman *et al*., 2013; Weber *et al*., 2001). In those cells, vemurafenib and dabrafenib were found to transactivate RAF dimers and lead to paradoxical activation of downstream ERK signaling (King *et al*., 2013; Poulikakos *et al*., 2010). Hence, vemurafenib and dabrafenib are selective toward B‐RAF^V600E^ mutant tumors and have no antitumor activity in *K‐RAS* mutant cancers.

BGB‐283 is a novel inhibitor against RAF family kinases, including WT A‐RAF, B‐RAF, C‐RAF, and B‐RAF^V600E^ (Tang *et al*., 2015). BGB‐283 is currently under Phase 1 clinical investigations (Desai *et al*., 2016b). Previously, we have shown that BGB‐283 induced significantly less paradoxical activation of MAPK signaling than vemurafenib in *K‐RAS* mutant cells, indicating that BGB‐283 may function against RAF dimers (Tang *et al*., 2015). In this report, we showed that BGB‐283 had potent inhibition of both WT B‐RAF and C‐RAF at high ATP concentration (1 mm), which is representative of the cellular ATP concentrations. Interestingly, the inhibition was also shown to be very time‐dependent. In comparison, vemurafenib showed minimal inhibitory effect on WT B‐RAF at physiologically relevant ATP concentration. The potent inhibitory activity of BGB‐283 against both RAF isoforms contributed to its enhanced cellular activity against either B‐RAF/C‐RAF heterodimers in vemurafenib‐treated Calu‐6 cells or B‐RAF homodimers in p61‐B‐RAF^V600E^‐expressing SK‐MEL‐239 C4 cells. This RAF dimer activity could potentially translate into antitumor activity in tumors that develop resistance to first‐generation B‐RAF inhibitors through dimer‐related mechanisms or tumors with non‐V600E B‐RAF mutations.

Target inhibition of another RAS downstream effector MEK has shown limited effect in suppressing the progression of *K‐RAS* mutant tumors (Blumenschein *et al*., 2015; Caunt *et al*., 2015). Previously, it was reported that RAF–MEK–ERK kinase cascade integrated with negative feedback loops exhibits NFA‐like properties (Sturm *et al*., 2010). Such a biological NFA design could provide resistance to inhibition of single components within the NFA (Sturm *et al*., 2010). It was suggested that concomitant blockade of RAF and MEK could weaken the NFA effect. This hypothesis was supported by a synthetic lethal screen using siRNA in which simultaneous targeting of RAF and MEK was shown to lead to enhanced inhibition in *K‐RAS*‐mutated cancer cells (Lamba *et al*., 2014). In this study, we demonstrated that BGB‐283, a RAF kinase family inhibitor with RAF dimer inhibitory activity, strongly synergizes with MEKi in inhibiting the growth *K‐RAS* mutant cancer cells. In contrast, vemurafenib, which is selective toward B‐RAF^V600E^ monomer, showed no synergistic effect with MEKi in *K‐RAS* mutant cells. We also observed that the synergy effect was not observed in the all NSCLC cell lines tested, which may reflect the biological heterogeneity of *K‐RAS* mutant NSCLC. It is well documented that among the NSCLCs with *K‐RAS* mutations, there is a subgroup shown to be K‐RAS‐independent (Román *et al*., 2018). Interestingly, when tested for combination effect with multiple MEKi, BGB‐283 showed differential enhancement of the activity of different MEKi toward *K‐RAS*‐mutated cells. BGB‐283 displayed very prominent synergistic effects when combined with MEKi (e.g., selumetinib and PD‐0325901) that induced stronger feedback phosphorylation of MEK. Interestingly, there seems to be less combination synergy when BGB‐283 combines with MEKi (e.g., trametinib, RO5126766) that induced less feedback phosphorylation of MEK despite the same combination trends being observed in all MEKi combinations. Previously, it was reported that MEKi including selumetinib and mirdametinib (PD‐0325901) increase the physical interaction between activated RAF and MEK proteins, rendering the latter less sensitive to MEKi (Fig. S2B) (Lito *et al*., 2014). In comparison, trametinib and RO5126766 single treatment induce minimum MEK phosphorylation through RAF reactivation, due to either disruption of RAF–MEK complexes or blockade of RAF phosphorylation site on MEK (Lito *et al*., 2014). BGB‐283 did not show obvious combination effect with these two MEKi, probably due to less dependency of RAF activities of pathway activation upon MEKi treatment. In accordance with their model, the combination of BGB‐283 and selumetinib showed better and more durable inhibition of MAPK signaling in *K‐RAS* mutant cells. In our studies, we show that BGB‐283 does not disrupt the RAF/MEK complex formation; instead, it promotes RAF–MEK complex formation. Meanwhile, BGB‐283 retains its ability to inhibit MEK phosphorylation through dimer inhibition (Fig. S2B,C).

The synergistic antiproliferative effect of combined BGB‐283 and selumetinib *in vitro* could further translate into enhanced antitumor effect and pathway suppression in preclinical xenograft models that harbor *K‐RAS* mutations. In both Calu‐6 NSCLC and HCT116 CRC xenograft models, combination treatment led to 88–100% partial and CR of tumors. It was reported that combination of B‐RAF and MEKi yielded better antitumor effect alone with less skin toxicity in both animal models and clinical studies against *B‐RAF* mutant tumors (Flaherty *et al*., 2012; Gadiot *et al*., 2013). The strategy for combining a RAF dimer inhibitor with a MEK inhibitor to maximize the inhibition of MAPK signaling of *K‐RAS* mutant tumors has certain similarities and appears attractive. In summary, this study provides evidence for combining clinical stage RAF dimer and MEKi to achieve enhanced therapeutic activities in *K‐RAS* mutant tumors, warranting evaluation of coblockade of RAF dimers and MEK to maximize the clinical benefit to *K‐RAS* mutant tumor patients. A clinical study evaluating this strategy using the combination of lifirafenib and mirdametinib is currently enrolling patients (NCT03905148).

## 5 Conclusions

K‐RAS is one of the intractable pharmacologic targets for human cancers. We find that RAF dimer inhibitor and MEK inhibitor show synergistic effect both *in vitro* and *in vivo* in *K‐RAS* mutant tumors and correlate the synergy with the PD effect. These findings add further evidence to support the strategy of combining RAF dimer and MEKi in the clinic for treating *K‐RAS‐mutated* cancer patients, which could lead to sustained MAPK pathway inhibition and overcome the resistance.

## Conflict of interest

The authors declare no conflict of interest.

## Author contributions

ZT, SC, YL, MW, CZ, WL, and LL conceived and designed the research. XY, RD, ZY, XZ, JW, YZ, YD, XH, WG, YL, and YG performed the experiments. XY, ZT, ZY, SC, ZC, ZH, MW, CZ, and LL analyzed the data and interpreted results of experiments. XY, LL, SC, ZY, PDS, and NR drafted, reviewed, and/or revised the manuscript.

## Supporting information


**Fig. S1.** Combinatorial effect of BGB‐283 and selumetinib on the proliferation of cells without MAPK pathway abnormalities.
**Fig. S2.** BGB‐283 induces RAF/MEK complex and inhibits MAPK pathway in Calu‐6 cells.
**Fig. S3.** The effect of BGB‐283 on body weight of nude mice subcutaneously implanted with human cancer xenografts.
**Table S1.** Antiproliferative effect by combining BGB‐283 with different MEKi in *K/N‐RAS*‐mutated NSCLC and CRC cells.
**Table S2.** Antitumor efficacy of BGB‐283 and selumetinib alone or in combination in human Calu‐6 NSCLC and HCT116 CRC xenografts.

**Appendix S1.** Statistical modeling and inference for EOHSA.
