# Supplementary material for: RAF dimer inhibition enhances the antitumor activity of MEK inhibitors in K‐RAS mutant tumors
Source: Mol Oncol. 2020 May 18;14(8):1833–49. doi: 10.1002/1878-0261.12698 (PMC7400788; doi:10.1002/1878-0261.12698)
Supplement: Supplementary file 1 — Fig. S1. Combinatorial effect of BGB‐283 and selumetinib on the proliferation of cells without MAPK pathway abnormalities. Fig. S2. BGB‐283 induces RAF/MEK complex and inhibits MAPK pathway in Calu‐6 cells. Fig. S3. The effect of BGB‐283 on body weight of nude mice subcutaneously implanted with human cancer xenografts. Table S1. Antiproliferative effect by combining BGB‐283 with different MEKi in K/N‐RAS‐mutated NSCLC and CRC cells. Table S2. Antitumor efficacy of BGB‐283 and selumetinib alone or in combination in human Calu‐6 NSCLC and HCT116 CRC xenografts. [file MOL2-14-1833-s001.pdf]

Supporting Information

Table S1. Antiproliferation effect by combining BGB-283 with different MEK inhibitors in *K/N-RAS* mutated NSCLC and CRC cells.

(A) BGB-283 and PD-0325901 synergistically inhibited the proliferation of multiple NSCLC cell lines harboring *K/N-RAS* mutations.

Cell Line	EOHSA		Maximum EC_{50} shift
	p-value*	Percentage**	for PD-0325901
NSCLC:			
**Calu-6**	0.0001	0.17	**59 fold ↓**
**A549**	<0.0001	0.17	**11 fold ↓**
**NCI-H2122**	<0.0001	0.16	**21 fold ↓**
**NCI-H23**	<0.0001	0.22	**22 fold ↓**
**SW1573**	<0.0001	0.27	**97 fold ↓**
**NCI-H358**	0.2361	0.00	18 fold ↓
**NCI-H1299**	<0.0001	0.14	**16 fold ↓**
**Calu-1**	0.8944	0.00	No shift
**Sk-Lu-1**	0.0015	0.09	**32 fold ↓**

(B) BGB-283 and pimarsertib synergistically inhibited the proliferation of multiple NSCLC and CRC cell lines harboring *K-RAS* mutations.

Cell Line	EOHSA		Maximum EC_{50} shift
	p-value*	Percentage**	for pimarsertib
NSCLC:			
**Calu-6**	<0.0001	0.42	**12 fold ↓**
**A549**	<0.0001	0.30	**42 fold ↓**
**NCI-H2122**	<0.0001	0.09	**6 fold ↓**
**NCI-H23**	<0.0001	0.44	**20 fold ↓**
**SW1573**	<0.0001	0.25	**＞100 fold ↓**
**NCI-H358**	0.0001	0.41	**>100 fold**
**NCI-H1299**	<0.0001	0.27	**45 fold ↓**
**Calu-1**	0.0001	0.11	**7 fold ↓**
**Sk-Lu-1**	<0.0001	0.17	**9 fold ↓**
CRC:			
**LS174T**	<0.0001	0.16	**15 fold ↓**
**Lovo**	<0.0001	0.19	**18 fold ↓**
**T84**	<0.0001	0.27	**44 fold ↓**
**DLD-1**	<0.0001	0.36	**＞100 fold ↓**
**HCT8**	<0.0001	0.12	**54 fold ↓**
**HCC2998**	<0.0001	0.2	**31 fold ↓**
**SW480**	<0.0001	0.17	No shift

(C) No significant synergic effect was detected between BGB-283 and RO5126766 in inhibiting the proliferation of *K/N-RAS* mutant NSCLC cells.

**Cell Line**	**EOHSA**		**Maximum EC_{50} shift for RO5126766**
	**p-value***	**Percentage****	
NSCLC:			
**Calu-6**	<0.0001	0.08	5 fold ↓
**A549**	<0.0001	0.16	*2 fold* ↓
**NCI-H2122**	0.0350	0.02	3 fold ↓
**NCI-H23**	<0.0001	0.20	4 fold ↓
**SW1573**	0.0005	0.12	5 fold ↓
**NCI-H358**	<0.0001	0.20	No shift
**NCI-H1299**	<0.0001	0.17	No shift
**Calu-1**	<0.0001	0.28	No shift
**Sk-Lu-1**	<0.0001	0.33	5 fold ↓

* p-values of Pacifico's approach for the hypothesis that at least one of the considered dose combinations has synergy per EOHSA.

** The percentage of the considered dose combinations with synergy found by Pacifico's approach at significance level 0.05.

No shift: less than 2 fold EC_{50} shift was detected by combining BGB-283 and various MEK inhibitors in indicated cell lines.

Table S2. Anti-tumor efficacy of BGB-283 and selumetinib alone or in combination in human Calu-6 NSCLC and HCT116 CRC xenografts.

Xenograft model	Dose of BGB-283 (mg/kg BID)	Dose of selumetinib (mg/kg BID)	N	Best Response Rate			TGI (day)
				PRR	CRR	ORR	
Calu-6	-	25	9	0%	0%	0%	93% (28)
	5	-	9	0%	0%	0%	78% (28)
	10	-	9	22%	0%	22%	97% (28)
	15	-	9	78%	0%	78%	>100% (28)
	2.5	25	8	88%	0%	88%	>100% (28)
	5	25	9	89%	11%	100%	>100% (28)
HCT116	5	-	8	0%	0%	0%	72% (21)
	-	25	8	13%	0%	13%	94% (21)
	5	25	8	88%	0%	88%	>100% (21)

PRR: partial response rate
CRR: complete response rate
ORR: overall response rate

Figure S1. Combinatorial effect of BGB-283 and selumetinib on the proliferation of cells without MAPK pathway abnormalities. Anti-proliferation effect of combining BGB-283 and selumetinib in (A) NCI-H209, (B) OUMS-23, and (C) HEK-293 cells was evaluated by EHOSA analysis. The considered dose combinations with synergy found by Pacifico's approach at significance level 0.05 are highlighted. (D) No significant synergistic effect between BGB-283 and selumetinib was detected from p-value, the percentage of dose combination with synergy and maximum EC_{50} shift for selumetinib in three cell lines.

Figure S2. BGB-283 induce RAF/MEK complex and inhibit MAPK pathway in Calu-6 cells. (A) Immunoblots probing for indicated proteins in Calu-6 cells transfected with indicated siRNAs and exposed to selumetinib. (B) Calu-6 cells were treated with BGB-283 and different MEKis for 3 h. Endogenous MEK1 was immunoprecipitated from cell lysates and RAF/MEK complexes were detected using immunoblotting. Whole cell lysates (WCL) were probed for indicated proteins related to MAPK pathway activation. (C) Calu-6 cells were treated with BGB-283 and vemurafenib (1, 3, 10 μM) for 1 h. RAF/MEK complexes were determined as in (B).

Figure S3. The effect of BGB-283 on body weight of nude mice subcutaneously implanted with human cancer xenografts. Body weights of mice bearing (A) Calu-6 NSCLC and (B) HCT116 CRC xenografts were measured twice weekly. Data are presented as average body weight ± SEM (N=9 for Calu-6, N=8 for HCT116 model).

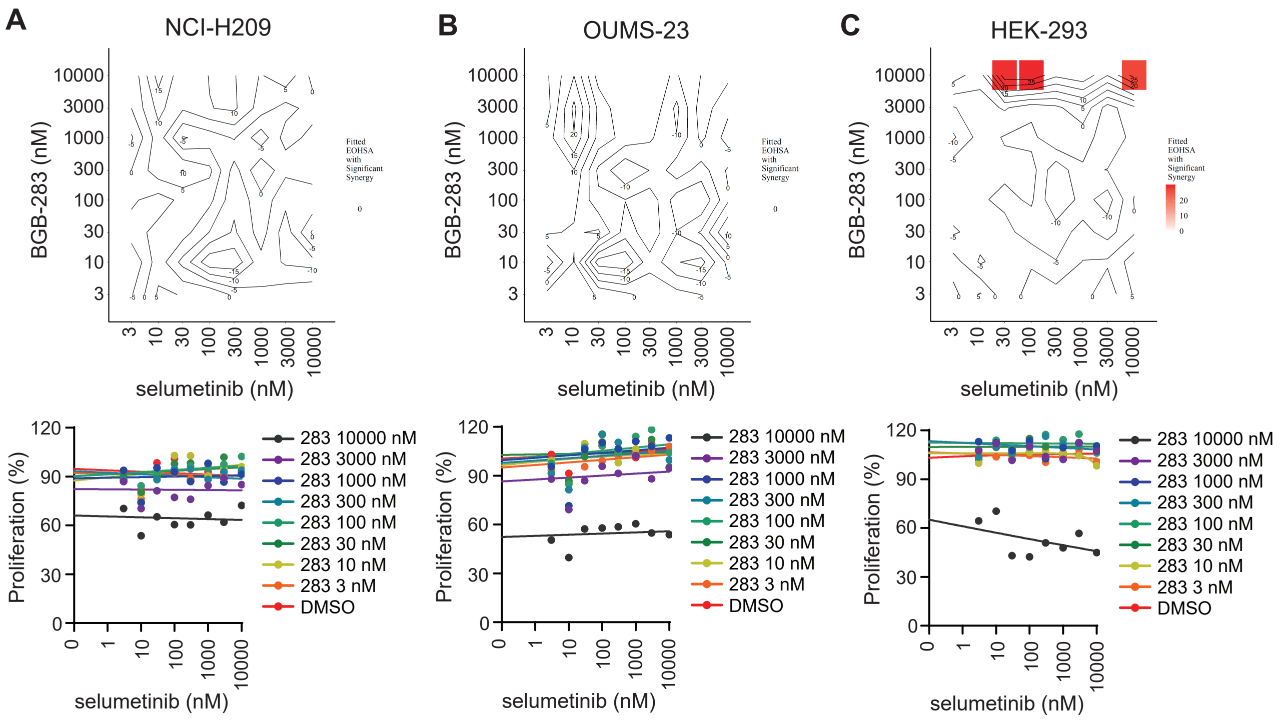

D

Cell line	EOHSA		Max EC_{50} Shift for selumetinib
	p-value*	Percentage**	
NCI-H209	0.5227	0.0	N/A
OUMS-23	0.6774	0.0	N/A
HEK-293	0.0062	0.046	N/A

* p-values of Pacifico's approach for the hypothesis that at least one of the considered dose combinations has synergy per EOHSA.
** The percentage of the considered dose combinations with synergy found by Pacifico's approach at significance level 0.05.

Figure S1

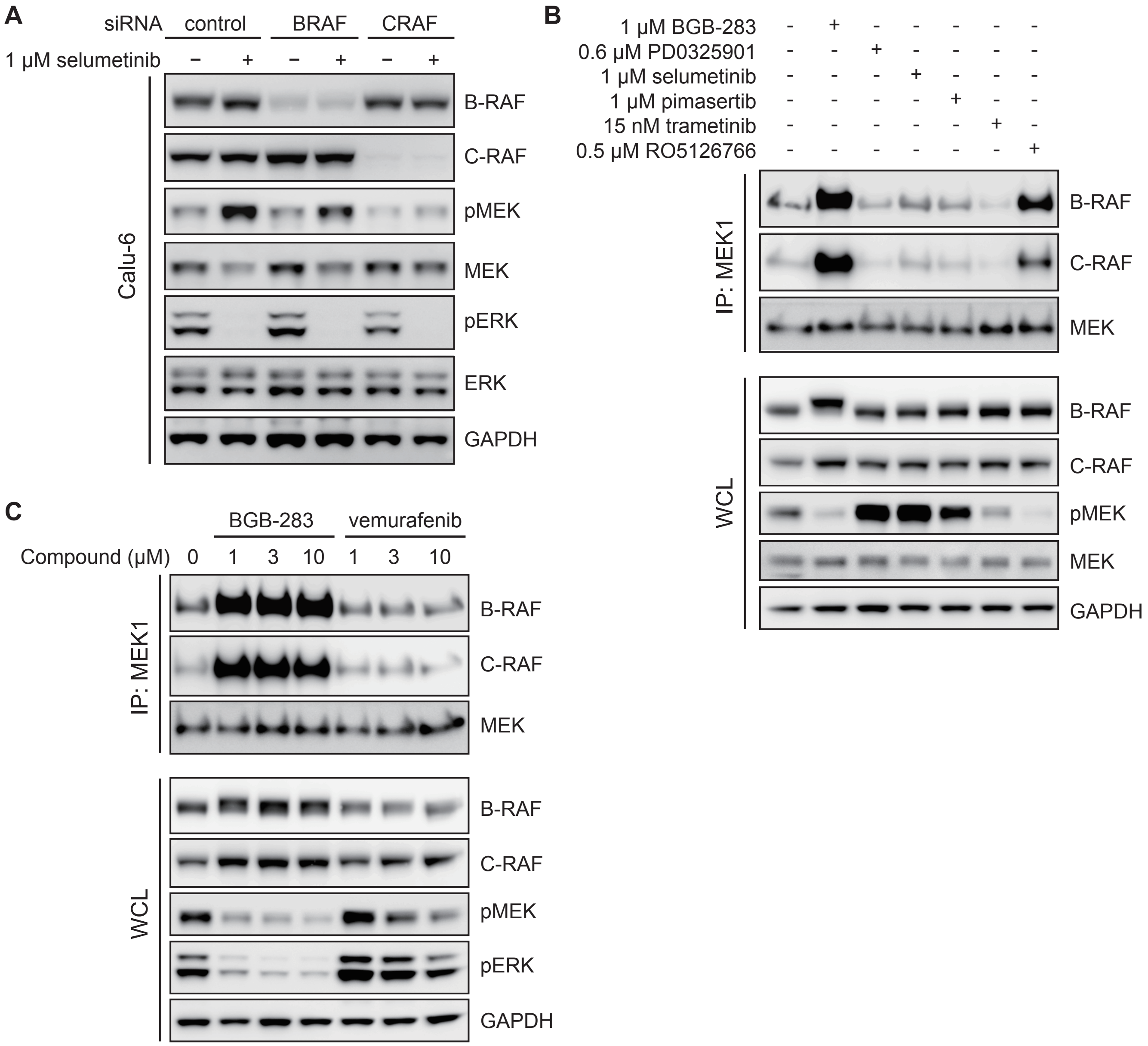
A
siRNA
control
BRAF
CRAF
1 μM selumetinib
−
+
−
+
−
+
Calu-6
B-RAF
C-RAF
pMEK
MEK
pERK
ERK
GAPDH
B
1 μM BGB-283
0.6 μM PD0325901
1 μM selumetinib
1 μM pimasertib
15 nM trametinib
0.5 μM RO5126766
IP: MEK1
B-RAF
C-RAF
MEK
WCL
B-RAF
C-RAF
pMEK
MEK
GAPDH
C
BGB-283
vemurafenib
Compound (μM)
0
1
3
10
1
3
10
IP: MEK1
B-RAF
C-RAF
MEK
WCL
B-RAF
C-RAF
pMEK
pERK
GAPDH

Figure S2

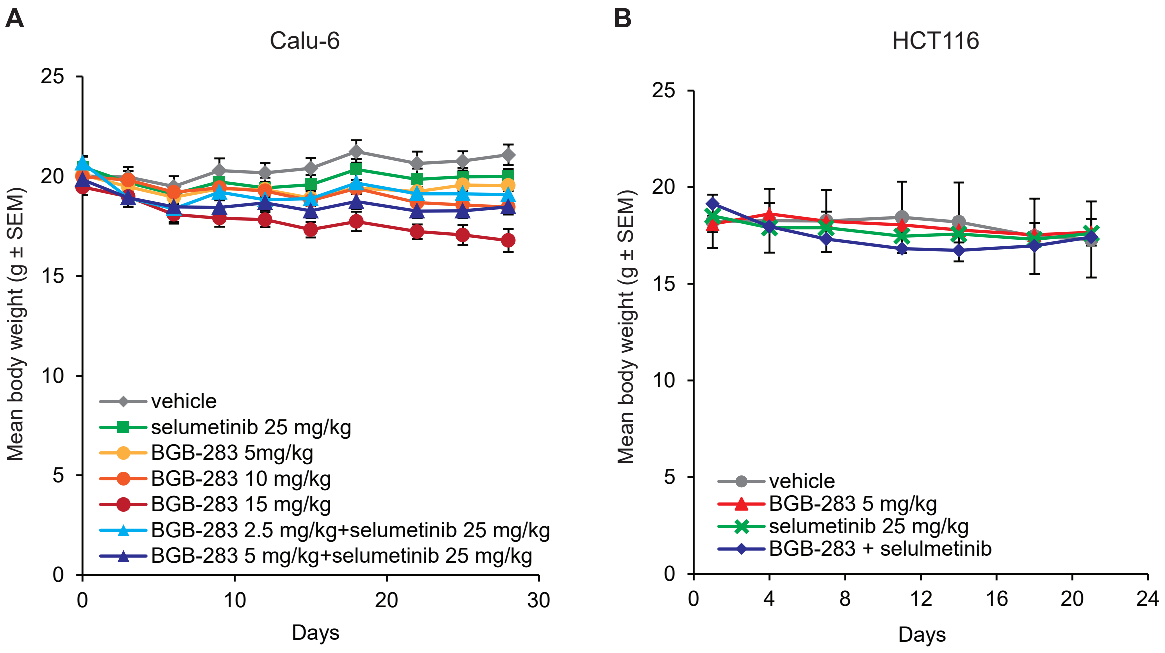
A
Calu-6
25
20
15
10
5
0
Mean body weight (g ± SEM)
0
10
20
30
Days
vehicle
selumetinib 25 mg/kg
BGB-283 5mg/kg
BGB-283 10 mg/kg
BGB-283 15 mg/kg
BGB-283 2.5 mg/kg+selumetinib 25 mg/kg
BGB-283 5 mg/kg+selumetinib 25 mg/kg
B
HCT116
25
20
15
10
5
0
Mean body weight (g ± SEM)
0
4
8
12
16
20
24
Days
vehicle
BGB-283 5 mg/kg
selumetinib 25 mg/kg
BGB-283 + selulmetinib

Figure S3
