# Supplementary material for: RAF dimer inhibition enhances the antitumor activity of MEK inhibitors in K‐RAS mutant tumors
Source: Mol Oncol. 2020 May 18;14(8):1833–49. doi: 10.1002/1878-0261.12698 (PMC7400788; doi:10.1002/1878-0261.12698)
Supplement: Supplementary file 2 — Appendix S1. Statistical modeling and inference for EOHSA. [file MOL2-14-1833-s002.pdf]

Supplementary Document

Statistical Modelling and Inference for EOHSA
in
RAF Dimer Inhibition Enhances the Antitumor Activity of MEK Inhibitors in K-RAS Mutant Tumors

February 11, 2020

1 Introduction

The aim of this document is to describe a model based synergy detection by EOHSA with false discovery control used in the paper "RAF Dimer Inhibition Enhances the Antitumor Activity of MEK Inhibitors in K-RAS Mutant Tumors".

2 Statistical Models for Raw

Let k be the number of replicates on the measurements of controls (positive or negative), $k = 1, \ldots, K$; and l be the number of replicates, $l = 1, \ldots, L$. Let (i, j) be the dose level combination, where $i = 1, \ldots, I$ and $j = 1, \ldots, J$. Further let Y_{kl}^N and Y_{kl}^P be the kth measurement of the negative and positive control at the lth replicate correspondingly, and Y_{ijl} be the measurement at dose combo (i, j) at the lth replicate.

For each l, let

$$\hat{\mu}_l^N = \frac{1}{K} \sum_{k=1}^{K} \log(Y_{kl}^N), \tag{1}$$

be the average of the negative control at the lth replicate. We assume that, for each i, j and l,

$$Z_{ijl} := \log(Y_{ijl}) - \hat{\mu}_l^N = \theta_{ijl} + \epsilon_{ijl}, \tag{2}$$

where θ_{ijl} represents the mean and ϵ_{ijl} is the residual. Meanwhile, we also have

$$Z_{kl}^P := \log(Y_{kl}^P) - \hat{\mu}_l^N = \theta_l^P + \epsilon_{kl}, \tag{3}$$

where θ_l^P represents the mean and ϵ_{kl} is the residual. We further assume that for each i, j, k and l, the residuals ϵ_{ijl} and ϵ_{kl} follow a normal distribution with mean 0 and variance σ_l^2 independent and identically.

2.1 Model Fitting

The unknown parameters are estimated as follows. For each i, j and l,

$$\hat{\theta}_{ijl} = Z_{ijl}. \tag{4}$$

For each l,

$$\hat{\theta}_l^P = \frac{1}{K}\sum_{k=1}^{K} Z_{kl}^P, \tag{5}$$

and

$$\hat{\sigma}_l^2 = \frac{1}{K}\sum_{k=1}^{K}(Z_{kl}^P - \hat{\theta}_l^P)^2. \tag{6}$$

It follows that $\hat{\theta}_{ijl}$ is normally distributed with mean θ_{ijl} and variance σ_l^2, and $\hat{\theta}_l^P$ is also normally distributed with mean θ_l^P and variance σ_l^2/K, where σ_l^2 can be consistently estimated by $\hat{\sigma}_l^2$ for large K.

2.2 From Raw to EOHSA

The EOHSA at dose combination (i, j) can be obtained as follows. For each l, the inhibition at (i, j) is

$$H_{ijl} = 1 - \frac{\theta_{ijl}}{\theta_l^P}. \tag{7}$$

At (i, j), the average inhibition is

$$A_{ij} = \frac{1}{L}\sum_{l=1}^{L} H_{ijl}. \tag{8}$$

Further the fitted EOHSA at (i, j), $i, j \geq 1$, is

$$\text{EOHSA}_{ij} = A_{ij} - \max(A_{0j}, A_{i0}) = \min(A_{ij} - A_{0j}, A_{ij} - A_{i0}). \tag{9}$$

The fitted EOHSA, denoted by $\widehat{\text{EOHSA}}_{ij}$, can be obtained from the same procedure by using $\hat{\theta}_{ijl}$ and $\hat{\theta}_l^P$. The distribution of $\widehat{\text{EOHSA}}_{ij}$ under the null is described in the Appendix.

3 Methodology

Consider the one-sided hypotheses

$$H_{0,ij} : \text{EOHSA}_{ij} = 0 \quad v.s. \quad H_{A,ij} : \text{EOHSA}_{ij} > 0 \tag{10}$$

for all (i, j), where $i, j \geq 1$. Let $S_0 = \{(i, j) : \text{EOHSA}_{ij} = 0\}$ and $S_1 = \{(i, j) : \text{EOHSA}_{ij} > 0\}$. Furhter, let R_T be the rejection region such that

$$R_T = \{(i, j) : \widehat{\text{EOHSA}}_{ij} \geq T\}, \tag{11}$$

where T is the threshold. The false discovery proportion for T is defined as

$$\Gamma(T) = \frac{\#(S_0 \cap R_T)}{\#R_T}. \tag{12}$$

The false discovery exceedance

$$\mathrm{FDX}_\gamma = \Pr\left(\Gamma(T) > \gamma\right). \tag{13}$$

To control the false discovery rate exceedance by α, i.e.,

$$\mathrm{FDX}_\gamma \leq \alpha, \tag{14}$$

we use the following approach as described in (Pacifico et al., 2004).

Firstly, we calculate the $(1-\alpha)$ confidence superset for S_0, denoted by U, meaning that

$$\Pr\left(S_0 \subset U\right) \geq 1-\alpha. \tag{15}$$

For all (i,j), $i,j \geq 1$, sort the fitted $\mathrm{EO\hat{H}SA}_{ij}$ in decreasing order and denote them by

$$\mathrm{EO\hat{H}SA}_{(1)} \geq \mathrm{EO\hat{H}SA}_{(2)} \geq \cdots \geq \mathrm{EO\hat{H}SA}_{(IJ)}. \tag{16}$$

For $r = 1, \ldots, IJ$, do the followings.

1. Set $U_r = \{r, \ldots, IJ\}$.
2. Compute

$$p(\mathrm{EO\hat{H}SA}_{(r)}, U_r) = \Pr\left(\sup_{r' \in U_r} \mathrm{EO\hat{H}SA}_{(r')} \geq \mathrm{EO\hat{H}SA}_{(r)}\right). \tag{17}$$

3. If $p(\mathrm{EO\hat{H}SA}_{(r)}, U_r) \geq \alpha$, then stop and set $U = U_r$; otherwise, increase r by 1 and go to Step 1.

Next, we select the threshold T as

$$T_\gamma = \inf\left\{t : \frac{\#(U \cap R_t)}{\#R_t}\right\}. \tag{18}$$

The resulting rejection region R_{T_γ} indicates the synergy area and

$$\Pr\left(\frac{\#(U \cap R_{T_\gamma})}{\#R_{T_\gamma}} \geq \gamma\right) \leq \alpha. \tag{19}$$

References

Pacifico, M. P., Genovese, C. R., Verdinelli, I., and Wasserman, L. (2004). False discovery control for random fields. *Journal of the American Statistical Association*, 99(468):1002–1014.

A Approximated Null Distribution

Consider the null hypothesis such that

$$H_0 : \text{EOHSA}_{ij} = \Delta_{ij} \quad v.s. \quad H_A : \text{EOHSA}_{ij} > \Delta_{ij}. \tag{20}$$

In this subsection, we approximate the distribution $\widehat{\text{EOHSA}}_{ij} - \Delta_{ij}$. Note that the result in this subsection only applies when σ_l^2 is small or large L.

For large K, the variance of $\hat{\theta}_l^P$ is comparable small to the one of $\hat{\theta}_{ijl}$. Therefore, it is treated as constant. It follows that, for each l,

$$\hat{H}_{ijl} - \hat{H}_{0jl} = \hat{\theta}_{0jl} - \hat{\theta}_{ijl}. \tag{21}$$

It follows that

$$\hat{H}_{ijl} - \hat{H}_{0jl} = \theta_{ijl} - \theta_{0jl} + \frac{\exp(\theta_{0jl})}{\exp(\theta_l^P)}\delta_{0jl} - \frac{\exp(\theta_{ijl})}{\exp(\theta_l^P)}\delta_{ijl}. \tag{22}$$

Let

$$\Delta_{ij} = \frac{1}{L}\sum_{l=1}^{L}\left(\exp(\theta_{0jl}) - \exp(\theta_{ijl})\right). \tag{23}$$

It leads to

$$\hat{A}_{ij} - \hat{A}_{0j} - \Delta_{ij} \approx \eta_{0j} \sim N(\mu_{0j}, \sigma_{0j}^2), \tag{24}$$

where

$$\sigma_{0j}^2 = \frac{1}{L}\sum_{l=1}^{L}\frac{\exp(2\theta_{0jl}) + \exp(2\theta_{ijl})}{\exp(2\theta^P)} \times \sigma_l^2. \tag{25}$$

For the same reason,

$$\hat{A}_{ij} - \hat{A}_{i0} - \Delta_{ij} \approx \eta_{i0} \sim N(\mu_{i0}, \sigma_{i0}^2), \tag{26}$$

where

$$\sigma_{i0}^2 = \frac{1}{L}\sum_{l=1}^{L}\frac{\exp(2\theta_{0jl}) + \exp(2\theta_{ijl})}{\exp(2\theta^P)} \times \sigma_l^2. \tag{27}$$

The correlation coefficient between $\hat{A}_{ij} - \hat{A}_{0j} - \Delta_{ij}$ and $\hat{A}_{ij} - \hat{A}_{i0} - \Delta_{ij}$ is

$$\rho_{ij} = \frac{1}{L}\sum_{l=1}^{L}\frac{\exp(2\theta_{ijl})}{\exp(2\theta^P)}\frac{\sigma_l^2}{\sigma_{0j}\sigma_{i0}}. \tag{28}$$

In sum, the test statistic $T.stat$ under the null could be approximated by the minimum of two normal random variables η_{i0} and η_{0j}, where $\min\{\mu_{0j}, \mu_{i0}\} = 0$, variance σ_{0j}^2 and σ_{i0}^2, and correlation coefficient ρ_{ij}. The p-value satisfies that

$$\text{p} = \Pr\left[T.stat \geq obs.value \mid H_0\right] \geq \Pr\left[T.stat' \geq obs.value \mid H_0\right] = \text{p}', \tag{29}$$

where $T.stat'$ is the minimum of the two normal random variables whose mean are 0 and variance σ_{0j}^2 and σ_{i0}^2, and correlation coefficient ρ_{ij}. The use of $T.stat'$

leads to a smaller Type I error and thus it leads to more conservative analysis under the alternative.

The p-value of $T.stat$ could be calculated through either the exact probability density function or the Monte-Carlo method

$$f_1(y) + f_2(y), \tag{30}$$

where

$$f_1(y) = \frac{1}{\sigma_{0j}} \times \phi\left(\frac{y}{\sigma_{0j}}\right) \times \Phi\left(\frac{\rho \times y}{\sigma_{0j}\sqrt{1-\rho^2}} - \frac{y}{\sigma_{i0}\sqrt{1-\rho^2}}\right) \tag{31}$$

and

$$f_2(y) = \frac{1}{\sigma_{i0}} \times \phi\left(\frac{y}{\sigma_{i0}}\right) \times \Phi\left(\frac{\rho \times y}{\sigma_{i0}\sqrt{1-\rho^2}} - \frac{y}{\sigma_{0j}\sqrt{1-\rho^2}}\right). \tag{32}$$
